# Royal Medical Society of Edinburgh

**Published:** 1837-04

**Authors:** 


### ROYAL MEDICAL SOCIETY OF EDINBURGH.

In another part of the present number, we have referred to this excellent and highly respectable Institution, the members of which resolved, on its completing its centenary, to celebrate the event by an appropriate festival. Accordingly, on Friday, the 17th February, this celebration took place, at the Hopetoun rooms. Owing to the season of the year, and the very general prevalence of the influenza, members from a distance were, for the most part, prevented from attending; nevertheless, one hundred and sixteen individuals assembled on the occasion. The proceedings commenced with the delivery, by Mr. Carpenter, senior president, of an oration, which was characterized no less by eloquence and power than by elegance of style. The chief object of the orator was to detail the leading features of the history of the Institution, and to point out the numerous advantages which had accrued to medical science from its establishment. We regret that our limits prevent us from giving any lengthened extracts from this discourse. We must, however, quote part of a passage relating to the connexion of some of our most distinguished men, in the early part of their career, with this Society; a circumstance extremely interesting in the literary history of our profession. We are much mistaken if the author of this oration is not himself destined to swell the list of those who, having received the benefit of an early stimulus from this Society, have repaid it, in after years, with the lustre of an enduring name.

"It is probably not generally known, that it was in the Medical Society that Dr. Crawford first promulgated his beautiful theory of animal heat, whose ingenuity none can deny, although the soundness of its premises may fairly be questioned; it has been characterized by the learned and philosophic Bostock as 'one of the most interesting and beautiful specimens of the application of chemical and physical reasoning to the animal economy that had ever been presented to the world.' Here, too, was commenced by the eminent author I have just quoted, that career of physiological enquiry and experimental research, by which he has earned a high and deserved reputation. The favorable reception of the dissertation read by Dr. Currie, on the effect of cold on the living animal body, must have prepared the way for those extended investigations, the publication of which ultimately led to a more correct appreciation of the therapeutic value of this agent. It was to this Society that Dr. Bateman presented his first essay in that branch of enquiry to which he afterwards so successfully devoted himself. It was here, too, that Dr. Henry exhibited his early attachment to the science of which he subsequently became so bright an ornament; and here also were commenced the beautiful researches of De la Rive on some of the nicest questions regarding caloric and magnetism. To the members of this Society were first communicated the result of those profound investigations into the physical history of mankind, which have given an European reputation to the name of Prichard. In our hall did the sagacity of Dr. Kay demonstrate the erroneous nature of the opinions of Bichat on the subject of asphyxia, and propound a theory deduced immediately from experiment, which is now received by our ablest physiologists; and there also took its rise the series of investigations into the atmospheric changes produced by animal and vegetable respiration, which have raised the name of Ellis to a high rank among the original enquirers in this department of science. . . . In the dissertations of Professor Jameson are to be found the earliest display of those talents which have been so successfully devoted to the advancement of a science whose gigantic strides and comprehensive grasp are constantly opening new fields of experimental enquiry, and affording new topics for philosophic speculation. The lectures of Dr. Thomson on Inflammation, and the work of Dr. Wilson Philip on the Phlegmasiæ, are among the results of debates in which the principal share was taken by these two individuals, whose well-earned reputation places them above any eulogy of mine. It must not be forgotten also, that the Elementa Medicinæ of Dr. Brown were published whilst their author occupied, for the third time, the chair of the Medical Society."

We cannot omit Mr. Carpenter's eloquent and energetic peroration:

"If in past times an ardent thirst after knowledge, a generous emulation in its acquirement, a steadfast pursuit of truth, a clear and unprejudiced judgment, were cherished in the breasts of the illustrious men who have shared our Association, by the opportunities of mental cultivation which they there enjoyed, and of which they have shown the abundant fruits, can we believe that these ennobling influences are no longer exerted, that from the increased fertility of the soil has resulted any diminution of its products? Gentlemen, let the memory of the energetic spirits, who, with Promethean skill, united the scattered elements into this giant frame, and animated it with living fire, not be without its effect this day, in stimulating us to carry on their noble purposes with renewed vigour. Their period of exertion has passed, and we have entered into their labours. But, though dead, they yet speak to us, in the dauntless courage with which they opposed the prejudices of the times, and the unwearied energy by which they succeeded in dispelling them. They speak to us in the noble reputation they have acquired for our institution, and which we are bound by the most sacred ties to maintain. They speak to us in the ardent zeal of their pursuit of truth, which they call upon us to imitate. They speak to us in the individual celebrity which they acquired, and encourage us to similar attainments. And could their spirits be with us this day, to witness the glorious result of their exertions, they would join with us in the earnest desire that the Society which they founded, and which now depends on us for its support, may still be the first in usefulness, as it was the earliest in formation; that as long as medical science shall continue to advance, and the pains of suffering humanity be assuaged by the healing art, so long it may persevere in its brilliant course, with undiminished lustre; and that many centenary gatherings of its members may unite, as we now do, in the fervent wish,—ESTO PERPETUA."

In the evening the members dined together, Professor Hope in the chair, assisted by the four presidents of the Society, Mr. Carpenter, Mr. Cormack, Dr. Charlton, and Mr. Bennet. Amongst the company present were many of the professors of the university and the principal medical gentlemen of eminence in the city of Edinburgh. We heartily wish this Society all the prosperity to which it is so well entitled and which it has so long preserved: if it is one of the most flourishing, it is certainly one of the most useful in the kingdom.

